# Implications of the COVID-19 pandemic on athletes, sports events, and mass gathering events: Review and recommendations

**DOI:** 10.1016/j.smhs.2023.07.006

**Published:** 2023-07-25

**Authors:** Jehad Feras AlSamhori, Mohammad Ali Alshrouf, Abdel Rahman Feras AlSamhori, Fatimah Maytham Alshadeedi, Anas Salahaldeen Madi, Osama Alzoubi

**Affiliations:** a School of Medicine, The University of Jordan, Amman, 11942, Jordan; b Medical Internship, Jordan University Hospital, The University of Jordan, Amman, 11942, Jordan; c Shmaisani Hospital, Amman, 11195, Jordan

**Keywords:** COVID-19, Cardiorespiratory, Athletes, Sport, Mass gathering events, Recommendations

## Abstract

Since the coronavirus disease 19 (COVID-19), which caused several respiratory diseases, was formally declared a global pandemic by the World Health Organization (WHO) on March 11, 2020, it affected the lifestyle and health of athletes, both directly through cardiorespiratory and other health related effects, and indirectly as the pandemic has forced the suspension, postponement, or cancellation of most professional sporting events around the world. In this review, we explore the journey of athletes throughout the pandemic and during their return to their competitive routine. We also highlight potential pitfalls during the process and summarize the recommendations for the optimal return to sport participation. We further discuss the impact of the pandemic on the psychology of athletes, the variance between the team and individual athletes, and their ability to cope with the changes. Moreover, we specifically reviewed the pandemic impact on younger professional athletes in terms of mental and fitness health. Finally, we shaded light on the various impacts of mass gathering events and recommendations for managing upcoming events.

## Highlights

• Cardiorespiratory system should be evaluated based on the clinical and symptomatic course and the severity of the illness.
• A cyclic approach is used for clinical evaluation, return to play (RTP) planning, and review (progress).
• Dynamic nature of return to play while paying continual attention to the athlete's growth and assessing for new problems.
• Athletes may develop psychiatric issues due to social exclusion, missed practices, monetary loss, and fear of infection.
• Maintaining proper personal hygiene and avoiding direct human interaction are crucial preventive strategies.

## Abbreviations

(CoVs): Coronaviruses
(SARS-CoV): Severe acute respiratory syndrome coronavirus
(MERS-CoV): Middle East respiratory syndrome coronavirus
(COVID-19): Coronavirus disease
(WHO): World Health Organization
(ARDS): Acute respiratory distress syndrome
(IOC): International Olympic Committee
(LGE): Late gadolinium enhancement
(ECG): Electrocardiogram
(RTP): Return to play
(MRI): Magnetic resonance imaging
(LV): Left ventricle
(HRQoL): Health-related quality of life
(hs-cTnT): High-sensitive cardiac troponin T
(CT): Computed tomography
(FeNO): Fractional exhaled nitric oxide
(CPET): Cardiopulmonary exercise testing
(6MWT): 6-min walking test
(sRPE): Session rating of perceived exertion
(FM): Fat mass
(FFM): Fat-free mass
(SOL): Sleep onset latencies
(TOD): Preferred training times
(TST): Total sleep time
(TIB): Time in bed
(PSQI): Pittsburgh Sleep Quality Index
(BMI): Body mass index
(MCS): Mental health component
(SF-36): Short-Form 36 Health Survey
(FIFA): International Federation of Association Football
(NBA): National Basketball Association
(NHL): National Hockey League
(UEFA): Union of European Football Association
(ACL): Anterior cruciate ligament
(BAU): Binding arbitrary unit

## Introduction

Coronaviruses (CoVs) are a group of highly diverse, enveloped, positive-sense, and single-stranded RNA viruses that can cause several diseases involving respiratory, gastrointestinal, and nervous systems with varying severity among humans and animals.^1^ Although most human coronavirus infections are mild, the last 20 years have seen two betacoronavirus epidemics, one involving the severe acute respiratory syndrome coronavirus (SARS-CoV)^2^, ^3^, ^4^ and the other involving the Middle East respiratory syndrome coronavirus (MERS-CoV)^5^^,^^6^ with fatality rates of 10% for SARS-CoV and 37% for MERS-CoV, which have generated more than 10 ​000 cumulative cases during the previous 20 years.^7^^,^^8^ The coronavirus disease 19 (COVID-19) is an infectious disease caused by the SARS-CoV-2 virus, which led to several respiratory ailments.^9^ On March 11, 2020, the World Health Organization (WHO) formally proclaimed COVID-19 a global pandemic with *extremely high risk.*^10^ The highly contagious character of the virus is primarily related to the ease with which it may spread from person to person, both via respiratory droplets and direct contact from surfaces to mucosal membranes.^11^ Most symptoms of SARS-CoV-2 infection, such as fever, cough, sore throat, fatigue, and the sequelae of pneumonia and acute respiratory distress syndrome (ARDS), are related to the respiratory tract.^9^

The COVID-19 pandemic has forced the suspension, postponement, or cancellation of most professional sporting events around the world in order to reduce the risk of viral propagation, with the question now being raised of how athletes can safely return to traditional competitive sports, taking into consideration the world's evolving knowledge of the virus and the shifting of governmental and public health recommendations.^12^ The virus caused social restrictions that led to the cancellation of sporting events in Brazil in March 2020, followed by outlawed sports training.^13^ In addition, on March 30, 2020, the International Olympic Committee (IOC) and the Japanese government announced that the 2020 Tokyo Olympics had been moved to July 20, 21^14^ This social isolation increased the likelihood that athletes would experience addictions, anxiety, sadness, and other mental health issues,^15^ and it also caused changes in eating practices during the pandemic.^16^ Moreover, numerous behavioral changes, including social withdrawal, staying in, avoiding crowds, and avoiding familiar exercise locales like gyms and health clubs, have been brought on by the COVID-19 pandemic.^17^ Teams like volleyball, football, field hockey, and cheer/dance groups had a higher incidence risk of infection among young athletes and students than those involved in individual sports like golf, tennis, and cross-country running.^18^ A report was carried out by Teran et al., which explained how a COVID-19 outbreak took place among a men's soccer team in Chicago. According to the report, the outbreak was likely due to the proximity of the team members during their meetings and training sessions that took place indoors.^19^ Additionally, an article by Dyke et al. on the COVID-19 outbreak among a university's men's and women's soccer teams noted that close contact during indoor practice and living in shared dorm rooms may have aided the outbreak.^20^ Accordingly, nonrestricted indoor contact and close proximity between sport team members may be significant factors aiding the spread of COVID-19.Furthermore, in badminton, a significant decline was noted in the athletes' levels of physical activity, with an increase in their idle time.^21^ It is essential to point out that incidence rates can differ even within sports teams; young volleyball club players reported an incidence rate of 7.9 per 100 ​000, compared to 1.7 per 100 ​000 in soccer.^22^ Positive test results were found in 25% of college athletes who were quarantined; the percentages were highest in football (54.4% COVID-19 positive), followed by swimming and diving (6.8% positive), and volleyball (5.5%).^23^ Softball, rowing, gymnastics, and golf had the lowest infection rates (1.1%–1.5%), while women had lower infection rates than men.^24^

The lockdown restrictions were put in place to stop the disease's spread and protect people and communities; however, this interfered with many daily activities, such as sports and general physical exercise. As a result, the likelihood of athletes experiencing adverse, acute, and possibly long-term health effects has increased due to these radical lifestyle changes brought on by self-isolation and physical inactivity. In addition, there is growing concern that athletes' immune systems and physical and mental health may decline due to a lack of exercise regimens and participation in sports activities, possibly causing the escalation of already-existing issues or the emergence of new diseases.^25^^,^^26^ In light of that, this review investigates cardiac, respiratory, and psychological considerations and their particular impacts on youth athletes. In addition, it provides recommendations for the upcoming future mass gathering events, especially sports tournaments.

## Impact of COVID-19 pandemic on cardiovascular health and return to play

Since the cases were reported, elevated levels of cardiac biomarkers have been noticed and linked to a worse outcome. Individuals with an elevated cardiac troponin level over the 99th percentile are more likely to need mechanical ventilation or even pass away than those with normal troponin levels.^23^, ^24^, ^25^, ^26^ One of the first studies on the clinical characteristics of individuals hospitalized with confirmed positive COVID-19 tests found that 12% had elevated troponin levels, suggesting cardiac injury.^27^ Regarding athletes who were complaining about flu-like symptoms, myocarditis has always been one of the most possible complications of viral infections. Even though the diagnosis is dependent on histological confirmation or late gadolinium enhancement (LGE) on cardiac MRI or myocardial oedema, myocarditis may be another factor contributing to the heart damage caused by COVID, according to recent case studies.^26^^,^^27^ The prevalence of myocarditis-related arrhythmias caused by the virus is unknown; however, COVID-19 infection increased out-of-hospital cardiac arrests in the general population by 50%.^28^ The results are descriptive and do not necessarily indicate an increased risk of sudden cardiac death or arrhythmias in otherwise "healthy" COVID-19-positive people.^28^

A committee of international experts classified athletes with COVID-19 infection into five categories: asymptomatic, mildly symptomatic, moderately symptomatic, severely symptomatic without mechanical ventilation, severely symptomatic with mechanical ventilation, and/or cardiac damage.^25^^,^^29^^,^^30^ They have also offered detailed advice on how to resume exercising safely for each group.^25^^,^^29^^,^^30^ Most COVID-19-infected athletes either show no symptoms or have minor symptoms; however, a thorough clinical evaluation is advised before they resume exercise. For asymptomatic athletes who tested positive for COVID-19, two weeks of self-isolation and rest from activity have been recommended. After that, a gradual return to exercise under the supervision of a medical team has been suggested.^31^ A more involved investigation process is advised for athletes who need hospitalization due to moderate-to-severe symptoms. In addition, athletes who have been hospitalized due to cardiac injury or respiratory symptoms should undergo a thorough multidisciplinary evaluation before beginning an exercise program.^25^^,^^29^^,^^30^

Many athletes are suspected of being infected, and only a small number of people will have had their COVID-19 infection verified by testing.^32^ Therefore, we advise a thorough clinical assessment that includes a medical history and physical examination for patients who have fully healed even without experiencing any new cardiovascular symptoms (symptom-free at rest for seven days and no earlier than day 10 from the start of symptoms). The initial assessment should determine whether any of the following symptoms are present: chest pain, dyspnea, palpitations, exertional vertigo, syncope, tachycardia, additional heart sounds, basal crepitations, or decreased air entry. A 12-lead electrocardiogram (ECG) and echocardiography are two more cardiac tests that should be performed before return to play (RTP).^32^ Before COVID-19, epidemiologic studies suggested that viral myocarditis in adults occurred at a frequency of 1.0–2.2 per 1 ​000 ​000 per year, making it a crucial concern. Therefore, a cardiac MRI is utilized to rule out myocarditis,^33^ followed by cardiopulmonary exercise testing and a 24-h Holter ECG as two secondary tests.^32^ Athletes with ongoing COVID-19 symptoms may need more than 14 days to recover. Wilson et al. advised a thorough history and physical examination, a 12-lead ECG, and cardiac magnetic resonance imaging (MRI) to search for myocarditis specifically. Cardiopulmonary exercise tests and a 24-h Holter ECG should be carried out if the MRI is normal. This stringent procedure ensures that athletes still sick are not pressured to exert themselves to the fullest before initial investigations are finished.^32^ It has been recommended by Wilson et al.^32^ that athletes who did not show any signs or symptoms of COVID-19 infection during the pandemic skip additional cardiac testing before deciding to RTP.^32^ Athletes with COVID-19 symptoms severe enough to require hospitalization should have a 24-h Holter ECG, 12-lead ECG, cardiac MRI, and cardiopulmonary exercise testing.^32^

Additionally, the international expert panel advises that cardiac troponin level be done only in athletes with persistent myocarditis-related symptoms and impaired myocardial function, as shown by imaging tests.^32^ The athlete should rest for at least 48 ​h before the investigation because intense exercise has been linked to a brief rise in blood cardiac troponin levels.^32^ However, routine troponin examination in all athletes who may have had COVID-19 infection is not advised because there are no recognized cut-off values for COVID-19 cardiac involvement, and most athletes will not have baseline troponin levels accessible during normal health.^32^ Patients who suffer from severe COVID-19 infection and who exhibit any positive findings should be treated similarly to other non-COVID-19 myocarditis patients, and additional testing, including cardiac MRI and implantable loop recorders should be carried out. If myocarditis or myopericarditis is confirmed, a period of disqualification of three to six months is necessary, depending on the clinical severity and duration of the ailment. It is safe to resume training and competing again if the left ventricular systolic function has returned to normal, serum myocardial injury biomarkers have stabilized, and 24-h Holter monitoring and exercise testing show no clinically significant arrhythmias, such as frequent or complicated repetitive ventricular or supraventricular arrhythmias.^34^

**Fig. 1:**
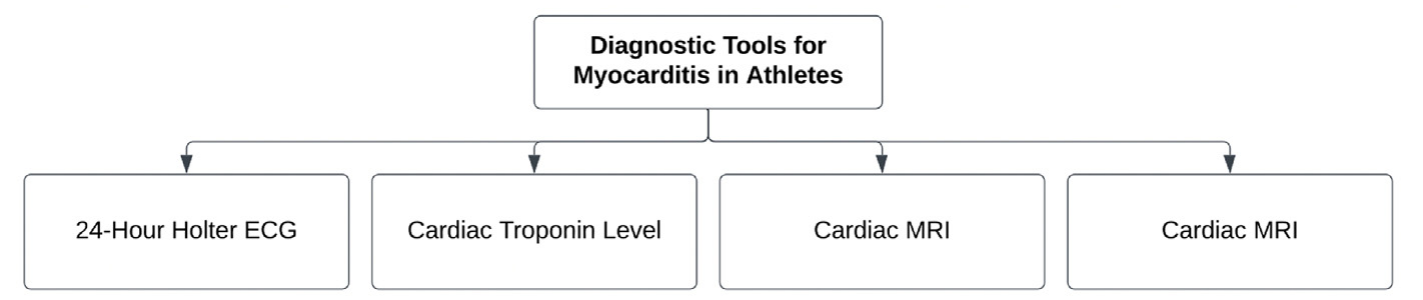
Diagnostic tools for athletes with suspected myocarditis. ECG, electrocardiogram; MRI, Magnetic resonance imaging.

Official athletic participation guidelines should be revised if clinical investigations reveal myocarditis. According to recommendations, athletes with established myocarditis should skip training sessions for 3–6 months to help achieve biological and clinical remission. The current professional consensus advises treating patients with proven myocarditis according to accepted standards. There is currently no proof that COVID-19-associated myocarditis is clinically distinct from other myocarditis types and requires a particular treatment plan.^35^, ^36^, ^37^ If left ventricular (LV) function and serum biomarkers have returned to normal and there are no symptoms or clinically significant arrhythmias on ECG exercise stress tests and ECG ambulatory monitoring, in this case, training can continue after the limitation period (3–6 months). In rare circumstances, parameter normalization can happen in as short as 3–6 months. An earlier return to play may be considered in some circumstances, but this guideline (3–6 months) is arbitrary.^35^, ^36^, ^37^ Additionally, it is essential to consider an athlete's mental state; even without a referral for a cardiology exam or potential sports restrictions, a COVID-19 diagnosis alone may substantially impact a person's psychological well-being.^38^^,^^39^ Fig. 1 summarizes the most important diagnostic tools for myocarditis in athletes.

Returning to exercise after recovering from a COVID-19 infection might be challenging. When athletes of all levels return to exercise after a viral infection, they should exercise with greater intensity and volume to regain their performance. In this case, return to exercise should be guided by clinical symptoms, with individuals urged to resume a low-intensity exercise program during self-isolation after 72 ​h of symptom remission. This program may be adapted to the equipment available, such as a stationary bike or treadmill, and can even incorporate resistance exercises.^25^^,^^40^ Since it increases the risk of upper respiratory tract infection and other serious illnesses, as well as the possibility of cardiac arrest, high-intensity exercise should be avoided. According to previous research, low-to moderate-intensity exercise boosts immune function, whereas high-intensity exercise suppresses the immune system and increases athletes’ susceptibility to illness.^26^^,^^31^^,^^41^^,^^42^

In addition to what Wilson et al.^32^ recommended, Hughes et al.^43^ suggested first engaging in low-intensity activity for one week before beginning more demanding and strenuous forms of exercise. Due to the lengthy time of inactivity, those who are recovering from an infection may be more susceptible to harm and injury since they will have had at least two weeks of rest without exercising due to their infection.^43^ Hughes et al., moreover, recommended a few strategies for returning to the sport after COVID-19. The severity of the symptoms is the most crucial factor to consider; in which they recommended a gradual recovery; those with no or moderate symptoms expect the resumption of pre-infection activity patterns in 7–14 days; those experiencing severe symptoms should be cautious and refrain from engaging in intense physical exercise.^43^ Those who are still asymptomatic or have had their symptoms relieved or reduced can gradually resume pre-infection activity levels. Exercise can be resumed depending on the individual's previous activity patterns.^43^

To begin with, individuals should begin by attempting 15–30 ​min of exercise at approximately 50% of their pre-infection intensity. If the procedure goes well, it should be repeated for two days (days 2–3). If all goes according to plan, the level of exercise can be increased to roughly 75% of what the person was used to before the illness started on day 4.^43^ The duration might be increased to at least 30 ​min. It should be repeated for two days if this is well tolerated (days 5–6).^43^ If there were no adverse effects from the attempted activity on day 7, the person might resume regular exercise routines from before COVID-19.^43^ A doctor should be contacted for a prospective heart assessment, which may involve an ECG, cardiac biomarkers, and echocardiography if a person has ongoing difficulty exercising at a level comparable to before infection 30 days after infection. However, it is still conceivable to have a reduced capacity for exercise as part of long COVID or post-COVID-19 syndrome, even in the setting of a cardiac workup that is entirely normal.^43^^,^^44^

Competitive athletes and student-athletes are only two of the many subpopulations that may face high degrees of anxiety, despondency, and terror, given the global mental health crisis accompanying this pandemic. Therefore, to decrease the overall mental health burden on athletes returning to play, an adequately implemented shared decision-making process is advised, along with the integration of psychological follow-up and intervention when necessary, particularly for COVID-19-positive athletes who are prohibited from participating.^36^^,^^45^, ^46^, ^47^

## Impact of the COVID-19 pandemic on respiratory health

Lung injury is anticipated to take place after COVID-19 infection. In prior pandemics, including SARS-CoV and influenza A virus (H1N1), many patients complained of chronic dyspnea, pulmonary function impairments, and a decline in health-related quality of life (HRQoL).^48^ Exercise-induced dyspnea, the recent onset or persistence of a post-COVID-19 cough and wheeze, and chest tightness that may or may not be associated with exertion are chronic post-COVID-19 respiratory symptoms. Airway diseases are prevalent in athletic populations; studies consistently show that one in every four endurance athletes has airway dysfunction, for example, exercise-induced bronchoconstriction due to asthma, which frequently goes undetected and is only discovered during screening assessments.^49^^,^^50^ Athletes did not need to undergo special respiratory testing if they did not exhibit any COVID-19 symptoms or signs during the outbreak.^32^ However, it should be emphasized that more than 50% of COVID-19 pneumonia patients who did not need hospitalization still had symptoms and decreased lung function after two months, according to Trinkmann's recent review article.^51^

Furthermore, most hospitalized patients with COVID-19 pneumonia experienced symptoms for 3–4 months^52^; according to a study by Garrigues et al., 42% of these patients experienced dyspnea, showing that lung impairment lingers long after the actual sickness has gone.^52^ The necessity of pulmonary follow-up in patients who had been hospitalized was the subject of a Swiss study.^53^ According to the study's findings, patients with COVID-19 who are hospitalized should have a pulmonary follow-up within three months after the onset of symptoms.^53^ In addition, a professional respiratory assessment should be obtained before RTP for every athlete who was hospitalized with radiologically confirmed COVID-19 pneumonia and dyspnea.^32^ The cardiovascular and respiratory systems are put through additional testing if dyspnea is still present at the first follow-up appointment.^54^

Before RTP, Wilson et al.^32^ advised that any athlete admitted to the hospital with radiologically confirmed COVID-19 pneumonia and dyspnea receive a specialist respiratory examination. This procedure is likely to require: (1) scheduled repeat imaging, (2) baseline physiological tests, including measurements of gas transfer and lung volume, and (3) cardiopulmonary exercise testing with oxygen analysis.^32^

A thorough evaluation should be performed on athletes who experience persistent respiratory symptoms caused by COVID-19 that take longer than 14 days to resolve to rule out the possibility of thromboembolic events, persistent intrapulmonary pathology, or heart damage.^32^ This examination should also include a chest X-ray, ECG, pulmonary function tests, and the measurement of inflammatory biomarkers such as C-reactive protein, high-sensitive cardiac troponin T (hs-cTnT), and D-dimer, if not already done.^32^ In addition, a thorax computed tomography (CT) scan is recommended when there is a high probability of thromboembolic or intrapulmonary abnormalities because it is the ideal imaging technique to detect post-COVID-19 aberrations and pulmonary vascular pathology (e.g., thromboembolism). A cardiac activity test with O_2_ saturation should be conducted, preferably with blood gas monitoring, if the etiology of the dyspnea is still uncertain. If there are desaturation episodes during exercise and a normal CT scan, consider getting a ventilation-perfusion scan to look for possible microemboli.^32^ If spirometry shows signs of an obstructive airway condition, a new diagnosis of asthma or post-infectious bronchial hyperreactivity should be investigated before choosing a course of treatment.^32^ It is crucial to evaluate the reversibility of bronchodilators as well as the existence of airway inflammation using fractional exhaled nitric oxide (FeNO), for example.^32^ For athletes who have experienced significant respiratory issues that require hospitalization, a complete respiratory assessment is also necessary before beginning RTP. Chest imaging and other tests, including troponin and D-dimer, are almost definitely already being performed in this situation, and therapy will be personalized based on the findings and the patient's course of recovery.^32^

**Fig. 2:**
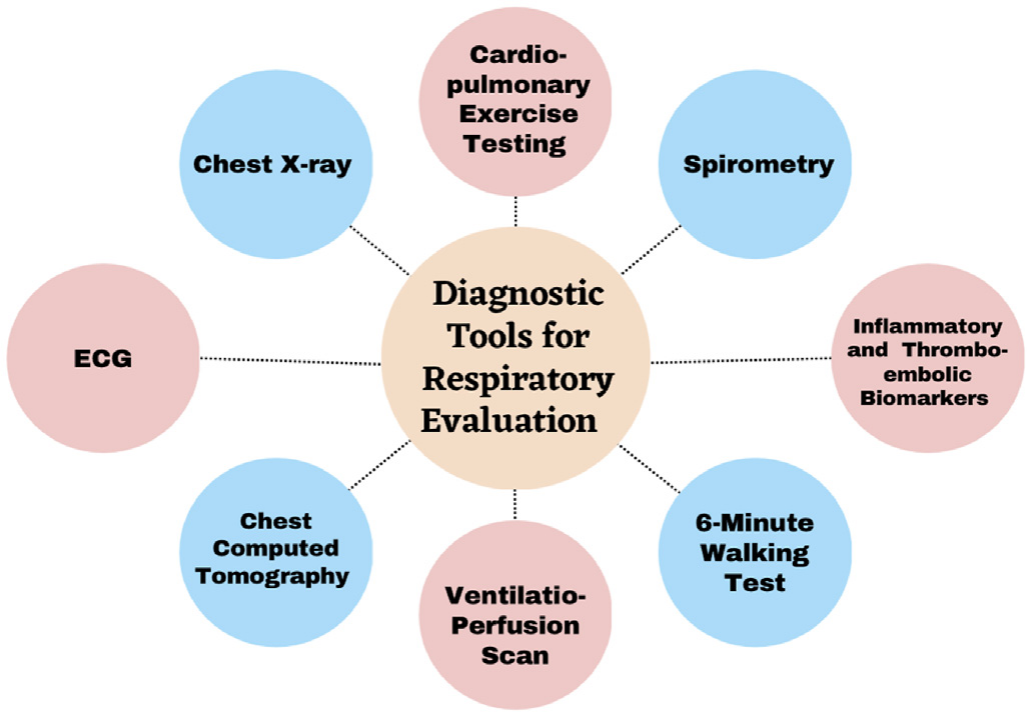
Diagnostic tools for athletes with respiratory symptoms. ECG, electrocardiogram.

A study by Moulson et al. on young athlete ranged between 18 and 35 years old with cardiopulmonary symptoms found that cardiopulmonary exercise testing (CPET) was clinically helpful in this population as a diagnostic tool to provoke the persistent or late-onset exertional cardiopulmonary symptoms in the cases of all normal testing results.^55^ CPET also identified around 42% incidence of abnormal spirometry and low breathing reserve; these findings suggest a potential therapeutic target and alternative explanation for the symptoms.^55^ In addition, alleviation of post-COVID-19 symptoms was associated with normalizing CPET measurements. Fig. 2 summarizes the most important diagnostic tools for athletes with respiratory symptoms.

The 6-min walking test (6MWT) is regularly used to test individuals after COVID-19 to detect exercise-related hypoxia and quantify reduced exercise capacity, in addition to all of the medical tests mentioned above, even though it has not been validated in COVID-19-associated pulmonary impairment.^56^A significant disadvantage of the 6MWT is that it is relatively easy to perform in the cases of highly fit people (i.e. athletes); so this test might not be of sufficient intensity to detect abnormalities in such population, where a more challenging test might be considered.^57^In addition, exercise testing is frequently used to look into respiratory impairment, and the actual 6MWT is a great way to evaluate various chronic respiratory conditions.^58^ Unfortunately, another study evaluating patients six weeks after severe illness found that 79% of participants' walking lengths during the 6MWT fell below the predicted value, even without desaturation.^59^

The national German S2k recommendations for managing COVID-19 patients who are hospitalized advise that, if feasible, a clinical follow-up beyond 8–12 weeks to identify any potential long-term effects should be done.^60^ Additionally, Wilson et al. suggested that athletes who suffered from mild to moderate COVID-19 symptoms (in which they treated their illness at home and recovered spontaneously), posed more of a challenge; as only few of them will have tested themselves and confirmed the COVID-19 infection, while most will simply suspect they had the infection and take precocious measures without definitively confirming the infection.^32^ In addition to that, a comprehensive clinical assessment that includes a medical history and physical exam is recommended for athletes who recovered entirely from the COVID-19 infection without any cardiovascular symptoms, defined as no symptoms for 7 days at rest.^32^ Moreover, any rehabilitation therapy must be gradual; thus, any deviation requires a “stop and evaluate” situation. Furthermore, athletes with asthma or other respiratory illnesses must be firmly encouraged to take their regular prescription respiratory medications precisely as instructed and doctors must discuss proper inhaler usage and spacing devices when necessary.^32^

## Impact of the COVID-19 pandemic on psychological health

Athletes may experience psychological illnesses due to social isolation, cancellation of training and tournaments, financial loss, and fears of infection or passing it on to others.^61^^,^^62^ Studies from several regions, including Europe,^63^, ^64^, ^65^, ^66^ Asia,^67^^,^^68^ Canada,^69^ South America,^70^ and the Middle East,^71^, ^72^, ^73^ showed that restrictions due to the pandemic had a significant negative impact on the mental health of athletes of all ages. A review that was conducted during the epidemic revealed that athletes of all levels, from amateurs to professionals, experienced various stresses, behavioral changes, and mental health problems.^74^

To better comprehend the situation's complexity from the athletes' point of view is to compare the pandemic experience to a sudden disease or injury. A sudden illness or injury causes sportspeople to drastically alter their sporting schedules, including career disruption and training partners, among other things.^75^ Exercise has been shown to decrease signs of anxiety and depression, primarily via promoting adult neurogenesis in the dentate gyrus of the hippocampus.^76^ It is worth noting that athletes reported lower levels of anxiety, melancholy, and posttraumatic stress disorder than healthy non-athletes.^71^^,^^73^ Epidemiological data from the COVID-19 pandemic show a positive and direct association between weekly exercise levels and mental health.^77^ Thus, engaging in physical activity or maintaining activity patterns throughout periods of forced restriction may reduce psychological stress. In contrast to non-athletes, student-athletes were still a part of a built-in community. In light of this, student-athletes may have had the edge over non-student-athletes in terms of socialization, even with virtual and isolation measures.^74^

The physical impacts of exercise and neurobiological mechanisms can be used to explain the putative mechanism of social restriction to distressing psycho-social symptoms in athletes. Exercise also develops behavioral mechanisms of change, such as self-control and self-efficacy.^78^ The athlete's social limitations may include social isolation, career instability, the unpredictability of the qualifying process, irregular and limited access to suitable training environments, and training partners. In terms of autonomy, incentive, and importance (or, more generally, the athletes' main competition), the postponement of the Tokyo 2020 Olympic Games represents a severe setback for the athletes' careers.^79^

Numerous athletes lost interest in the sport and stopped competing after stopping their training during their one-year COVID-19 social limitation. Additionally, a study conducted by Demarie, S.^80^ found that six weeks after training resumed, Horse-riders' performance in subsequent eventing competitions decreased because of the eight weeks of training restrictions and competition avoidance brought on by the COVID-19 sanitary emergency, with the Dressage test being the discipline most negatively impacted.^80^ In addition, anxiety and session rating of perceived exertion (sRPE) were substantially correlated with performance in dressage, supporting the idea that cognitive discomfort contributes to how hard a person works and how well they execute in such an emotionally charged discipline.^80^ The shorter Show-jumping and Cross-country courses in the post-lockdown Eventing competitions allowed RPE to remain stable, sRPE to significantly reduce, and cardiovascular strain to remain stable at pre-lockdown values for all three types of tests, in accordance with the guidelines for returning to training and competition after the COVID-19 emergency.^80^ It is also important to realize that the pandemic had differing effects on athletes of various ages, sports, and countries. In Italy, 50% of young individuals, 32% of adolescents, and 30% of adult athletes reported feeling anxious, showing that younger people had reduced stress tolerance.^65^ The epidemic significantly impacted Romanian Olympians, kayakers/canoeists, track and field athletes, tennis players, and footballers.^64^ These results may indicate that it was more difficult for individual athletes than team athletes to deal with pressures during the lockdown.^65^ However, in a study by Silva et al., where they followed 23 young badminton athletes, including one group who stopped their daily training routine for 8 months (retrained) and the other group who stopped for one year (detrained), no significant difference between groups was found for any of the emotional state variables examined, including tension, despair, fury, energy, fatigue, and confusion.^81^ This implies that the primary outcomes of the long-term COVID-19 social restriction impact primarily the body composition, including fat mass (FM) and fat-free mass (FFM), of young badminton athletes. However, over 52% of athletes reported feeling awful during the lockdown,^82^ and athletes from less resource-rich developing countries were more mentally affected than athletes from more resource-rich ones.^83^

The lockdown caused top athletes' insomnia and sleep quality to worsen; this was mainly due to longer sleep onset latencies (SOL), later bedtimes, preferred training times (TOD), and daytime naps. Additionally, elite athletes slept more overall (TST) and in bed (TIB) while in lockdown. Compared to top athletes who reduced their training intensity, those who maintained their training level during lockdown experienced less insomnia and better sleep quality. The lockdown length negatively impacted athletes’ sleep-wake patterns and training, with longer lockdowns being linked to more insomnia and less restful sleep.^84^ Overall, the lockdown negatively impacted all the evaluated sleep metrics. For instance, increases in Pittsburgh Sleep Quality Index (PSQI), SOL, later bedtime, and later preferred TOD to train were also observed when comparing pre-to-during-lockdown.^84^ It has been reported that many lockdown-induced behavioral changes could cause the observed disruption of training and sleep.^46^^,^^85^^,^^86^ Moreover, increased nighttime feeding, higher caffeine and alcohol intake, more prolonged and later daytime naps, have all been proven to contribute to prolonged SOL.^86^ Additionally, during the lockdown, elite and sub-elite athletes reported higher levels of anxiety, worry, and sadness, which can impair the quality of their sleep.^85^

## Impact of COVID-19 pandemic on youth athletes

Only a few studies have examined the effects of lockdowns on young professional soccer players. In a study that included eight professional youth soccer players aged 16 to 19 from a U19 Bundesliga team from Hesse, Germany, in 2020, they found that the COVID-19 lockdown caused a considerable increase in body weight, fat mass, and body mass index (BMI) following the home training phase.^87^ However, during the COVID-19 lockdown, the implemented at-home monitored training schedule effectively maintained muscle strength, endurance performance, and sleep quality. In addition, isokinetic strength and endurance performance showed no appreciable effects on physical performance before and after the home training period.^87^ Furthermore, interestingly, the mental health component (MCS) of the Short-Form 36 Health Survey (SF-36) showed that they had increased mental health after the lockdown; this might be explained by the fact that players' sleep habits remained unaffected (PSQI) and the obligation of school attendance was eliminated. Similar results were reported by Grazioli et al.^88^; however, they were on adults. They found that Brazilian professional soccer players' body weight and fat mass significantly increased after 63 days of home training compared to the regular off-season. The athletes were measured twice, once after 63 days of at-home training in May 2020 and once again 24 days after a typical off-season in December 2019. The players exercised at home during the lockdown, using nothing but their bodies as resistance. Soccer experts anticipated that the lockdown would have a negative effect on professional soccer players' physical abilities, but this was not the case.^88^ Despite the lockdown, youth professional soccer players’ cardiorespiratory fitness and endurance performance did not significantly change following COVID-19 home training. The COVID -19 outbreak has disrupted community-organized sports due to officials canceling, drastically reducing, or delaying athletic participation to stop viral propagation.^55^ Regarding worries about their psycho-social, physical, and professional growth, this significantly impacted young athletes and their families.

## Impact on mass gathering events and recommendations

### Mass gathering

According to WHO, a mass gathering is a planned or spontaneous event with a head count large enough that it can strain the planning and response resources of the health system in the community where it occurs. Examples on Mass gatherings include the International Federation of Association Football (FIFA) World Cup, Olympic Games, and other significant sporting events.^89^ Therefore, it poses a significant risk of importing or exporting infectious diseases. Viral transmission among attendees and the local population is a rising public health issue associated with large gatherings.^90^ It is crucial to understand that COVID-19 has infected individuals while traveling to another country, which may result in local and international transmissions of the disease.^91^ Because of this, mass gatherings could likely become potential super-spreaders of the pandemic.^92^ As mentioned previously, indoor activities during sport events or throughout team preparations and training sessions are suggested to drive greater viral transmission.^19^^,^^20^ Importantly, the risk of indoor transmission during sport events has been shown to be significantly affected by the implemented hygiene practice in which direct contact between participants along with ventilation and air exchange systems all play an important role in controlling indoor viral spread.^93^

### Sports events

Since late 2019, all sporting leagues and competitions around the globe have suffered the impact of the SARS-CoV-2 in one way or another. National Basketball Association (NBA), National Hockey League (NHL), Union of European Football Association (UEFA) Champions League, and Spain's "La Liga" are some examples of internationally renowned team sport competitions that were suspended. As a consequence of these government alterations and restrictions, almost all teams and athletes ceased their training activities, causing a negative impact on their performance capacity and, consequently, their professional careers. It is safe to say that both professional and amateur sports were affected by the constraints.^94^

According to an Australian poll, sports teams experienced the highest decline in participation during COVID-19.^95^ Sports were undoubtedly impacted in different ways and to different degrees according to the seasonality, nature of contact participation, and general age of participants. Pandemic recovery efforts need to be prioritized for those most affected. Men had a more significant decline in participation than women, mainly because men were more likely to engage in club-based competitive sports, which were outlawed by COVID-19 regulations. COVID-19 may be the “ideal storm” for sports and physical activity stakeholder groups to reevaluate and offer a broader range of programs to match a changing and diverse consumer demand.^95^ Moreover, the number of sports injuries, including Achilles tendon and hamstring tendon injuries, increased, while the number of anterior cruciate ligament (ACL) injuries remained steady between 2017 and 2019 and 2020 NFL seasons. Injury rates were consistently high during the first four games of the 2020 NFL season, despite being higher in the preseason in previous years.^96^

Robust infection control procedures must be implemented early on, and the decision to restart sporting events should be based on the local number of cases.^97^ Zheng et al. performed a meta-analysis on forty-three studies which investigated how exercising with various types of face masks impacts physiological and psychological factors.^98^ Their findings indicate that wearing face masks during exercise has a limited impact on gas exchange, pulmonary function, and psychological outcomes among healthy individuals. Moreover, exercise performance appears to be minimally affected using face masks.^98^

Safety is still crucial despite the importance of fitness and sports, especially for competitive players. To prevent the COVID-19 pandemic from spreading further, everyone should participate in safe sports and adopt the appropriate safety measures.^97^ Based on these first results, we hypothesize that facemask exercise increases physiological reactions due to restricted ventilation, heavier breathing, and sympathetic responses.^99^^,^^100^ Compared to those who did not wear masks, subjects who did so reported higher physiological demands (RPE ​= ​10.8 vs. 12.7 ​at 6 ​min). The Borg Scale classifies moderate-intensity exercise as "very tough" at an RPE of 12.7. Some volunteers who were wearing facemasks during the assessment reported having an uneasy feeling of dyspnea, which is consistent with past findings.^101^ Convection, evaporation, and radiation processes are hindered by skin irritation and moisture accumulation inside the masks, which negatively impact human thermoregulation's respiratory and cutaneous mechanisms.^97^ As a result of the increased cardiorespiratory strain following covert activity, it is imperative to exercise safely. It is often a good idea to stop when the heart rate reaches 150 beats per min or 70% of the age-predicted maximal heart rate.^97^^,^^102^ This is particularly true for older hikers and those with numerous coexisting medical conditions. As a result of the additional physiological demands on the body, exercises should be modified for each person's capacity when performed with a facemask. The usage of facemasks while exercising had no impact on physiological responses or exercise performance, according to a recent meta-analysis by Shaw et al.^103^ Furthermore, the study showed that exercising while wearing a mask has no effect on performance for people of various ages and health situations.^103^

COVID-19 is a highly contagious infection in sporting environments, especially in contact sports, due to its prolonged survival on surfaces, lengthy incubation period, and the various ways from which it is contacted and spread. It is also worth mentioning the amount of traveling and transportation required for sports teams which imposes additional risks of contracting and spreading the virus. Avoiding direct human contact as much as possible and maintaining good personal hygiene are essential preventive measures. In contact sports, where close contact with opponents is inevitable, athletes should avoid harmful on-field behavior to prevent unnecessary infection. Sports should consult with their medical advisors, an infectious disease specialist, and applicable local or state authorities to come up with a valid justifiable protocol to approve the reestablishment of daily sports events in their pre-COVID state.

We have recommended some procedures to control the situation during the COVID-19 pandemic and future mass gathering events like sport competitions as well as holidays, music and theater performances, popular religious practices, and other relevant gatherings of close contact with others.

### Recommendation

We have recommended some procedures to control the situation during the COVID-19 pandemic and future mass gathering events. Using a facemask when in contact with others is one of the most essential and fundamental precautions that must be highlighted, along with strict and routine hand cleanliness. Therefore, the general population needs to be reminded to routinely wash their hands with soap and water, as well as before and after using the restrooms and eating. In addition, the general people must learn to wash their hands for at least 20 ​s and to rub their hands with alcohol when they are not visibly dirty. Furthermore, facemasks could reduce the spread of aerosolized particles, particularly in high-density regions during mass gatherings.

For sports events, we recommend the following:

a. A certificate of vaccination indicating a second dose or booster dose within six months (the number of doses depends on how long the vaccine is practical and durable) or a certificate of recent COVID-19 infection (within the last six months: adaptive protective immunity following natural infection of SARS-CoV-2 may persist for at least six to eight months).^104^
b. Visitors must have a COVID-19 immunoglobulin G antibody titer of more than 33.8 binding arbitrary unit (BAU)/mL before departing (which indicates a protective immunity to COVID-19).^105^
c. A COVID-19 passport that lists prior virus exposure, testing results, and immunization status for participants, delegates, and spectators.^106^
d. Free COVID-19 rapid test sites are made available close to each place, and the results are verified in the COVID-19 passport and glocalization application for guests before they are admitted.^107^
e. A bubbling system reinforced by stringent public health rules remains the primary preventative measure for global athletes.
f. More medical facilities and emergency response strategies should be developed for the competition, and emergency response strategies should consider the probability of irregular rises in the number of cases.^108^
g. To better inform preventative strategies, real-time communication of the COVID-19 infection in the host countries and cities should be adopted.^108^
h. Despite being somewhat inconvenient, it has been demonstrated that wearing a mask, avoiding social situations, and keeping oneself clean all dramatically reduce COVID-19 transmission.^109^

Finally, for future holiday events, we suggest the following: It is acknowledged that maintaining a mask is challenging because many holiday activities focus on eating, which calls for removing the mask. However, other preventive measures, such as limiting big crowds and risky activities, will still be crucial in this situation.^110^ In addition, it is recommended that people self-quarantine if they have a suspected infection to make the annual holidays more secure.^111^

## Conclusion

As the cardiorespiratory system was considered in this evaluation based on the clinical and symptomatic course and the severity of the illness, it is essential to carefully analyze the potential cardiorespiratory effects of COVID-19. A cyclic approach is used for clinical evaluation, return to play (RTP) planning, and review (progress). This considers the dynamic nature of RTP while paying continual attention to the athlete's growth and assessing for new problems. Athletes may develop mental health limitations because of social exclusion, missed practices and competitions, monetary loss, and worries about becoming sick or infecting others. As noted, the pandemic-related restrictions severely affected the mental health of athletes of all ages. Athletes' sleep-wake habits and training were also significantly impacted by the lockdown duration, with longer lockdowns associated with increased insomnia and less restful sleep. Due to its persistence, protracted incubation time, and milder symptoms, COVID-19 is highly contagious in sporting settings, particularly in contact sports. Therefore, maintaining proper personal hygiene and avoiding direct human interaction as much as possible are crucial preventive strategies, and never to forget the importance of self-awareness in the unsteady global pandemic we still live in.

## Authors’ contributions

**Jehad Feras Samhouri:** Conceptualization, Literature search, Writing-Original Draft, Project administration, Writing - Review & Editing, and Supervision. **Mohammad Ali Alshrouf**: Writing - Review & Editing, Project administration, and Supervision. **Fatimah Maytham Alshadeedi**: Literature search, Writing - Original Draft. **Abdelrahman Feras Samhouri**: Literature search, Writing - Original Draft. **Anas Salahaldeen Madi**: Writing - Review & Editing, Creation of figures. **Osama Alzoubi**: Research idea and conceptualization, Writing - Review & Editing, and Supervision.

## Submission statement

All authors have read and agree with manuscript content. While this manuscript is being reviewed for this journal, the manuscript has not and will not be submitted elsewhere for review and publication.

## Conflict of interest

We wish to confirm that there are no known conflicts of interest associated with this publication and there has been no significant financial support for this work that could have influenced its outcome.
